# N-Butylidenephthalide Inhibits the Phenotypic Switch of VSMCs through Activation of AMPK and Prevents Stenosis in an Arteriovenous Fistula Rat Model

**DOI:** 10.3390/ijms21197403

**Published:** 2020-10-07

**Authors:** Hsin-Han Yang, Yue-Xuan Xu, Jie-Yi Chen, Horng-Jyh Harn, Tzyy-Wen Chiou

**Affiliations:** 1 Department of Life Science and Graduate Institute of Biotechnology, National Dong Hwa University, Hualien 974, Taiwan; 810413102@gms.ndhu.edu.tw (H.-H.Y.); 610813007@gms.ndhu.edu.tw (Y.-X.X.); 610613001@gms.ndhu.edu.tw (J.-Y.C.); 2 Bioinnovation Center, Tzu Chi Foundation, Buddhist Tzu Chi General Hospital, Tzu Chi University, Hualien 974, Taiwan; 3 Department of Pathology, Buddhist Tzu Chi General Hospital and Tzu Chi University, Hualien 970, Taiwan

**Keywords:** n-butylidenephthalide(BP), VSMCs phenotypic switch, PDGF, AMPK, mTOR, stenosis, arteriovenous fistula (AVF)

## Abstract

The phenotypic switch of vascular smooth muscle cells (VSMCs) plays a pivotal role in the development of vascular disorders, such as atherosclerosis, stenosis and restenosis, after vascular intervention. In our previous study, n-butylidenephthalide (BP) was reported to have anti-proliferating and apoptotic effects on VSMCs. The purpose of the current study is to further investigate its role in platelet-derived growth factor (PDGF)-induced VSMC phenotypic modulation in an arteriovenous fistula model. In vitro, we observed that BP inhibited the PDGF-induced cytoskeleton reorganization of the VSMCs. The enhanced expression of vimentin and collagen, as well as the migration ability induced by PDGF, were also inhibited by BP. By cell cycle analysis, we found that BP inhibited the PDGF-induced VSMCs proliferation and arrested the VSMCs in the G0/G1 phase. In an arteriovenous fistula rat model, the formation of stenosis, which was coupled with a thrombus, and the expression of vimentin and collagen in VSMCs, were also inhibited by administration of BP, indicating that BP inhibited the PDGF-induced phenotypic switch and the migration of VSMCs. Besides, the inhibitory effects of BP on the phenotypic switch were found to accompany the activated 5’ AMP-activated protein kinase (AMPK) as well as the inhibited phosphorylation of mTOR. Knockdown of AMPK by gene silencing conflicted the effects of BP and further exacerbated the PDGF-induced VSMCs phenotypic switch, confirming the modulating effect that BP exerted on the VSMCs by this pathway. These findings suggest that BP may contribute to the vasculoprotective potential in vasculature.

## 1. Introduction

The vascular smooth muscles (VSMCs) within the tunica media are characterized by remarkable phenotypic plasticity [1]. Upon injured stimuli, VSMCs undergo a “phenotypic switching” process from a contractile phenotype to a proliferative and migratory synthetic phenotype. The phenotypic switch of VSMCs is a fundamental step in developing vascular pathologies, such as neointimal hyperplasia and restenosis, after the intervention of percutaneous transluminal angioplasty (PTA) or the establishment of an arteriovenous fistula (AVF). The dedifferentiation of VSMCs can be stimulated by the released growth factors and inflammatory cytokines, such as PDGF, IFN-γ and TNF-α, after vascular injury. Of these, PDGF has been reported as the most potent mitogens that cause the dedifferentiation of VSMCs via activating variable intracellular signal pathways, including the PI3K/Akt, Rho and mTOR pathways [2,3]. Therefore, to inhibit the PDGF-stimulated VSMCs’ phenotypic switch may reduce the development of neointimal hyperplasia and stenosis.

N-butylidenephthalide (BP), a natural compound isolated from *Angelica sinensis*, has been investigated for a variety of pharmacological activities, such as antitumor growth, suppression of platelet aggregation, relaxation of vessels and inhibition of VSMCs proliferation [4,5]. In our previous study, we have proved that BP inhibited neointimal hyperplasia in a balloon-injured rat carotid artery due to its dual effects of anti-proliferative and apoptotic induction on VSMCs [6]. However, little is known about whether BP inhibits the PDGF-induced phenotypic switch of VSMCs as well as inhibiting the formation of stenosis in an arteriovenous fistula.

AMP-activated protein kinase (AMPK) is a conserved metabolic sensor that maintains the cellular energy by fine-tuning the ATP-reducing and ATP-synthesizing processes in all eukaryotic cell types [7]. Published studies indicate that activation of AMPK inhibits the cell proliferation in tumor cells, non-malignant cells as well as PDGF-induced VSMCs [8,9]. Its roles in regulating metabolism and contractile functions in skeletal and vascular tissues were also demonstrated by studies that used AMPK activators such as AICAR and metformin, indicating that AMPK might be a therapeutic target for vascular remodeling disorders like stenosis and restenosis [10,11].

Therefore, the purpose of this study is to determine whether BP inhibits the phenotypic switch of VSMCs both in vitro and in an arteriovenous fistula (AVF) rat model, as well as the effect on regulating AMPK, which can serve as a potential mechanism to modulate the phenotypic switch of VSMCs.

## 2. Results

### 2.1. n-Butylidenephthalide (BP) Inhibited the PDGF-Induced Vascular Smooth Muscle Cells’ (VSMCs’) Phenotypic Switch

**Figure 1:**
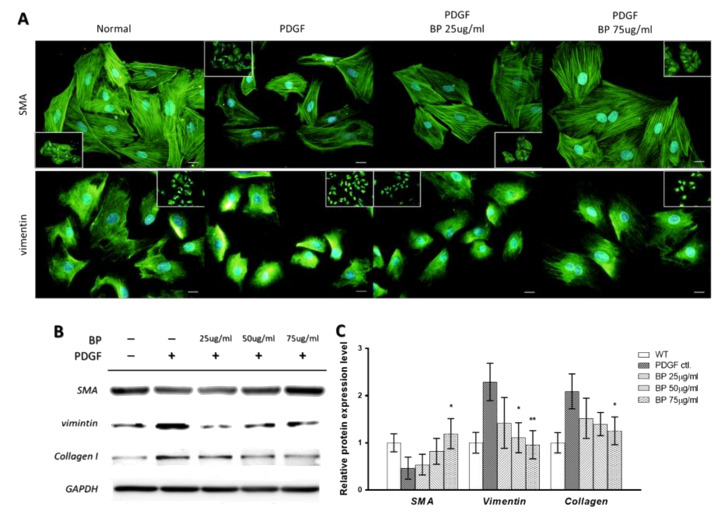
n-Butylidenephthalide (BP) inhibited PDGF-induced phenotypic switch. Measurements of morphology and protein expression in A7r5 treated with BP: (**A**) Representative images of PDGF-induced A7r5 in the absence or presence of BP (25 and 75 μg/mL) for 24 h. Scale bar = 50 μm; (**B**,**C**) Western blotting analysis of phenotypic marker expression. All data are presented as the means ± standard deviation of three independent experiments. * *p* < 0.05 and ** *p* < 0.01 versus the PDGF group.

To investigate whether BP modulated the VSMCs’ phenotype, we first examined the morphology and measured the molecular markers of contractile and synthetic VSMCs. We treated A7r5 cells with 10 ng/mL of PDGF-bb and different concentrations of BP (25, 50 and 75 µg/mL) for 24 h. The morphology and the relative protein abundance of A7r5 was examined by immunofluorescence and Western blotting, respectively. As shown in Figure 1A, the PDGF-treated A7r5 showed a marked phenotypic switch, as reflected by the disorganization of α-smooth muscle actin (SMA) and aggregation of vimentin, whereas the BP treatment inhibited the effect. Besides, the reduced α-SMA and enhanced vimentin as well as collagen protein level induced by PDGF were inhibited by the treatment of BP (Figure 1B). These results demonstrate that BP inhibits PDGF-induced phenotypic switch of VSMCs.

### 2.2. BP Inhibited the Functional Characteristics of the Synthetic VSMCs

**Figure 2:**
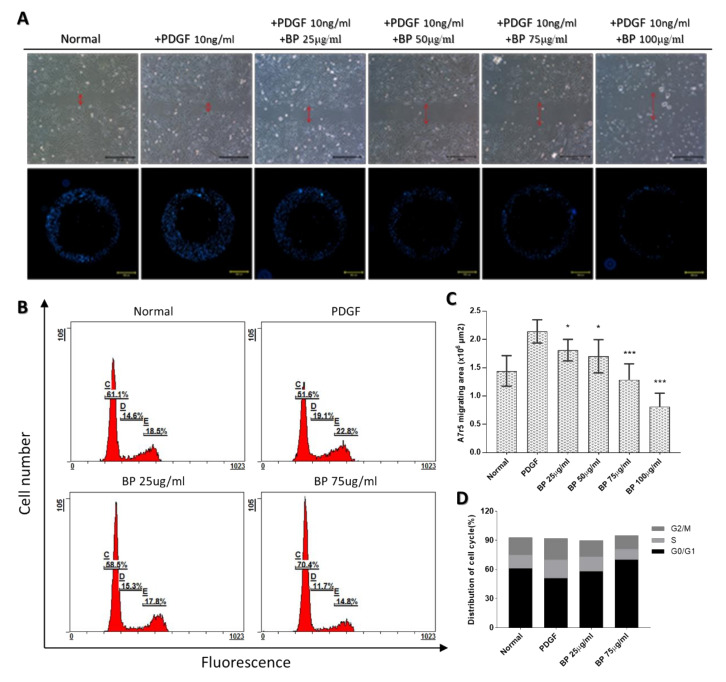
BP inhibits migration and arrests the PDGF-induced A7r5 in the G0/G1 phase in a dose-dependent manner. (**A**) A wound-healing assay (**upper** panel, scale bar = 500 μm) and Oris system migrating assay (**lower** panel, scale bar = 500 μm) were performed since A7r5 was treated with PDGF (10 ng/mL) and BP (0, 25, 50, 75 and 100 µg/mL) for 24 h; (**B**) Cell cycle analysis through PI staining and following flow cytometry for the A7r5 after 24 h of PDGF (10 ng/mL) and BP treatment (0, 25 and 75 ug/mL); (**C**,**D**) Quantification of the Oris system migrating assay and cell cycle analysis. Data are expressed as the mean ± SEM. * *p* < 0.05, *** *p* < 0.001.

The migration of synthetic VSMCs from the media to intima and its proliferation plays a major role in the progression of neointimal hyperplasia. To examine the anti-migrating effect of BP, the wound-healing and Oris assays were performed [12]. As shown in Figure 2A, the migrating ability of PDGF-induced A7r5 decreased with BP treatment in a dose-dependent manner. At a 50 μg/mL concentration of BP, PDGF-induced A7r5 exhibited a significantly delayed migration in both the wound-healing and Oris system analyses.

For examining the anti-proliferating effect of BP, a cell cycle analysis was performed. As shown in Figure 2B, the proportion of A7r5 in the G0/G1 phase was significantly decreased from 61.1% to 51.6%, and the proportion of A7r5 in the S phase was increased from 14.6% to 19.1% after the PDGF induction. Compared with the PDGF group, the BP treatment at 25 and 75 μg/mL, in which the dosage was lower than IC50 (100 ± 3 μg/mL), significantly increased the proportion of the G0/G1 phase and decreased the proportion in the S phase in a dose-dependent manner, suggesting that BP can dose-dependently promote A7r5 into a quiescent stage.

### 2.3. BP Inhibited Thrombosis, Neointimal Hyperplasia and Phenotypic Switch of VSMCs in an Arteriovenous Fistula (AVF) Rat Model

**Figure 3:**
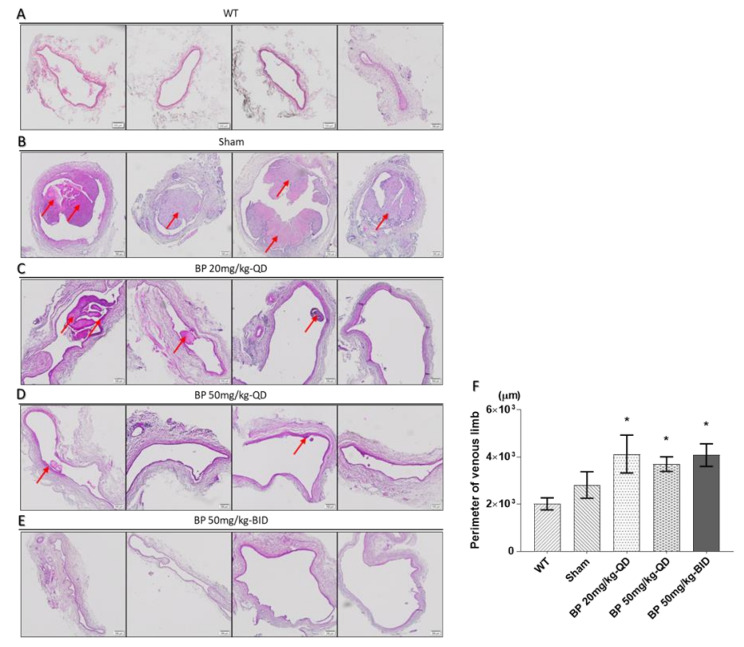
Histological staining of a venous limb of arteriovenous fistula (AVF) 30 days after establishment, and the comparison of lumen size among rats treated with different doses of BP. The thrombus and neointimal hyperplasia were indicated by a red arrow. (**A**) WT; (**B**) Sham; (**C**) 20 mg/kg-QD BP; (**D**) 50 mg/kg-QD BP; and (**E**) 50 mg/kg-BID BP. A significant reduction in thrombus and neointimal hyperplasia was observed in rats treated with BP. (**F**) Measurement of lumen size as reflected by the perimeter of the venous limb. Scale bar = 100 μm. Data are expressed as the mean ± SEM. *n* = 4 per group. * *p* < 0.05 versus sham.

To evaluate the anti-stenosis activity of BP in vivo, we established an AVF model on experimental rats. Rats were then randomized into control (*n* = 4) and BP oral-administered (20, 50 mg/kg-QD and 50 mg/kg-BID, *n* = 4 each) groups for 30 days. The venous limbs collected from the sacrificed rats were processed by H&E. Given that BP has been reported to have an antiplatelet effect in a previous study [4], we directly examined whether BP inhibited the formation of thrombosis and neointima hyperplasia in this study. As shown in Figure 3A–D, treatment of BP significantly inhibited the formation of thrombosis and neointimal hyperplasia in the 20 mg/kg-QD group. Besides, we observed a significant increase in the lumen size in the treatment groups, indicating that BP can benefit the maturation of AVF by removing the thrombosis (Figure 3E).

**Figure 4:**
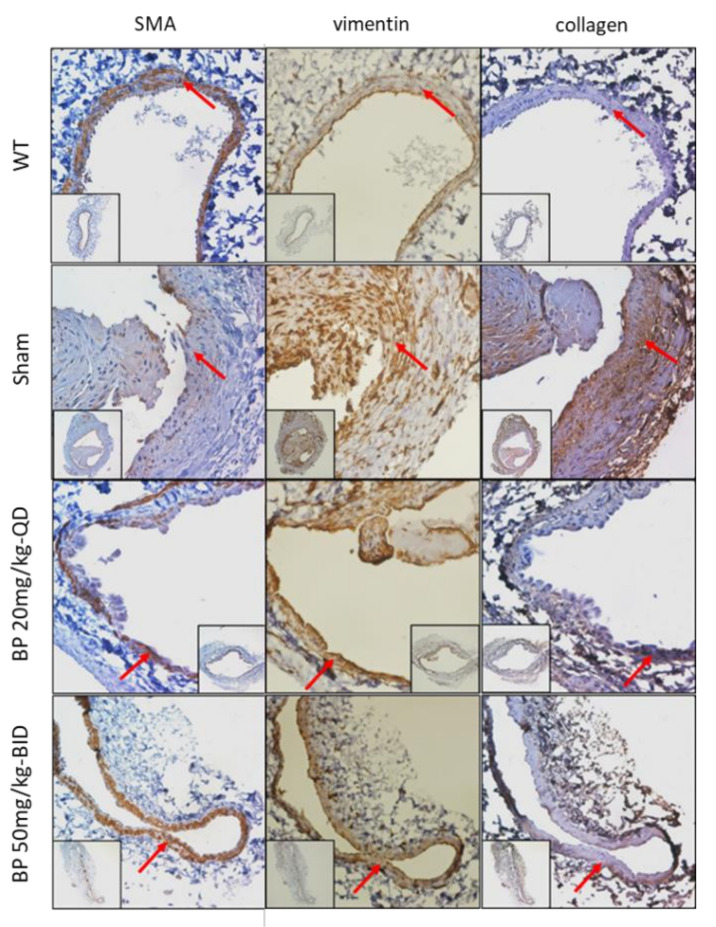
Immunohistochemical detection (brown color) of a venous limb of AVF from rats treated with different doses of BP for 30 days. The expression of a phenotypic-specific marker (SMA, vimentin and collagen I) in an adjacent section of each group is indicated by a red arrow. Significant inhibition of phenotypic switch was observed in rats treated with BP compared to the control (sham) group. Scale bar = 100 μm.

To examine whether BP inhibited the VSMCs phenotypic switch, the IHC staining of the contractile type marker SMA, as well as the synthetic marker vimentin and collagen, were performed in this animal study. As shown in Figure 4, the quiescent, contractile VSMCs in the wild type (WT) vein exhibited a high density of SMA, whereas the vimentin and collagen were expressed at a low density. Compared to the WT vein, the neointimal area in the venous limb of AVF in the control group exhibited higher vimentin and collagen, whereas the SMA was rarely expressed, indicated that the VSMCs underwent a phenotypic switch from the contractile to synthetic state. Because the neointimal area was rarely found in the BP treatment group, the media, the origin of the VSMCs, was examined instead. As expected, the VSMCs in the media exhibited a higher SMA and lower vimentin and collagen, suggesting that BP can inhibit the VSMCs phenotypic switch, thereby inhibiting the formation of neointimal hyperplasia.

### 2.4. BP Inhibited the PDGF-Induced VSMCs’ Phenotypic Switch through the AMPK/mTOR Signaling Axis

**Figure 5:**
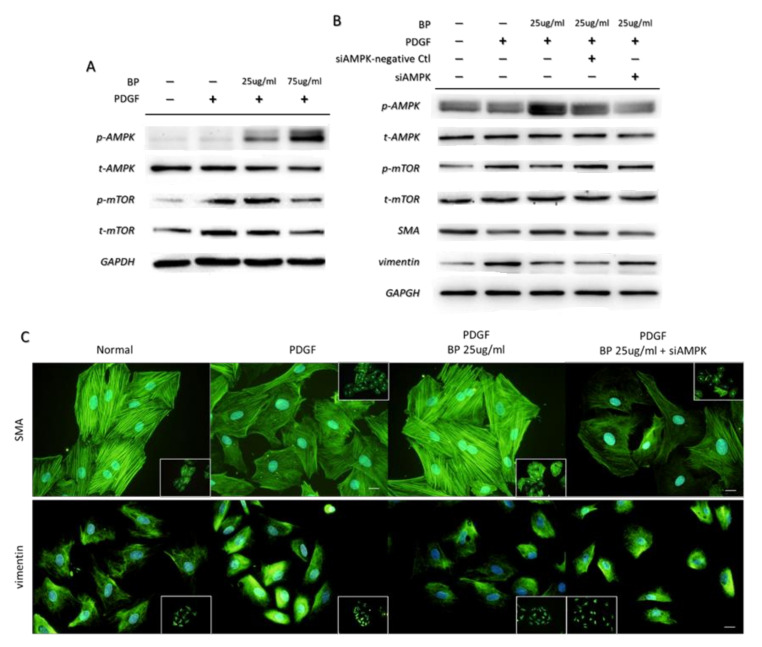
BP inhibited the PDGF-induced phenotypic switch by activating AMPK. (**A**) Protein expression of AMPK and mTOR after treatment of PDGF and BP for 24 h; (**B**) Protein expression of AMPK, mTOR and phenotypic markers in PDGF-induced A7r5 treated without or with BP in the absence or presence of siAMPK; (**C**) Representative images of PDGF-induced A7r5 treated without or with BP in the absence or presence of siAMPK. Scale bar = 50 μm

To illustrate the role of AMPK in the anti-phenotypic switching effects exerted by BP, we examined the expression level of AMPK and mTOR. We treated PDGF-induced A7r5 with BP for 24 h and demonstrated that the BP treatment induced AMPK and reduced mTOR activities, as measured by their phosphorylation (Figure 5A). To determine whether the activation of AMPK by BP inhibited functional changes associated with the synthetic VSMCs phenotype, we conducted an AMPK alpha knockdown on the A7r5. As shown in Figure 5B,C, the inhibition of AMPK abolished the effect of BP on PDGF-induced A7r5 as measured by the morphology, and by expression of SMA, vimentin. These results suggest that BP inhibits the phenotypic switch of VSMCs through activating the AMPK signaling axis.

## 3. Discussion

In our previous study, we have demonstrated that BP can inhibit restenosis in a rat balloon-injured carotid artery model through inducing the apoptosis directly on VSMCs. In the present study, we further explored the novel findings that the phenotypic switch of VSMCs, induced by PDGF, can be inhibited by BP—both in vitro and in an arteriovenous fistula rat model. In addition, these effects are found to be associated with AMPK activation, in that knocking down of AMPK conflicts the PDGF-induced VSMCs’ phenotypic switch. These findings provide new insights into BP as a novel system with significant therapeutic potential for vascular remodeling diseases.

To enlarge the lumen, arterialize the venous limb and provide adequate blood flow to achieve a functioning dialysis access are the core goals of AVF creation [13] In clinical settings, the primary failure due to the early thrombosis, the failure to mature due to aggressive neointima hyperplasia or impaired outward remodeling, as well as late failure due to the anastomotic configuration and repeated needle puncturing, are the three major mechanisms that leads to AVF dysfunction. Among the three mechanisms, the factors affecting early thrombosis are complex, including turbulent blood flow, dysregulated sheer stress and surgical injury, which lead to epithelial cell dysfunction and platelet activation. Based on previous studies [4] and our results, we speculate that BP is most likely to inhibit the cyclooxygenase (COX) expression in epithelial cells, as well as inhibit the collagen expression in VSMCs to sustainably inhibit the activation of platelets, thereby inhibiting the release of PDGF, which prevents the formation of stenosis. In addition, we found that the degree of lumen enlargement did not differ among each BP treatment group, indicating that the formation of a thrombus is the major factor affecting the lumen enlargement in the venous limb of AVF. Besides, we provide evidence that BP reduces neointimal hyperplasia in the venous limb of AVF by inhibiting the phenotypic switch and by enhancing the expression of the contractile protein of the VSMCs in the media of a vessel. These indicated that BP not only inhibit the PDGF-induced VSMCs’ migration from the media toward the intima, but also promotes the venous arterialization at a later stage of fistula development to withstand the impact of repeated cannulation in the hemodialysis treatment.

AMPK is a central cellular energy sensor that is present in eukaryotic cells. Its activation upon increased AMP/ATP and ADP/ATP ratios promote glucose transport and fatty acid oxidation, thereby maintaining the balance between energy supply and demand. Under metabolic stress, AMPK is also activated to suppress the anabolism to ensuring cell survival [7]. In a high-fat diet mouse model, the AMPK-KO VSMCs exhibit a synthetic phenotype and develop unstable plaque [14], whereas the treatment of AICAR promotes the contractile index in the injured VSMCs [10], emphasizing AMPK’s critical role, especially in VSMCs that exhibit a highly changeable energy turnover. In our present study, the effect of BP on activating AMPK was found in PDGF-induced VSMCs in the serum non-deprivation model. In addition, we observed that, in the serum deprivation model, the PDGF-induced VSMCs exhibited a large amount of apoptosis, and the treatment of BP further exacerbated it. These demonstrate that sufficient nutrients and the regulation of metabolism have higher priority than responding to the injured stimuli in VSMCs, although the stimuli promote the anabolism and the phenotypic switch of VSMCs. Besides, we found the inhibition of AMPK by pan-AMPK inhibitor compound C further enhanced the expression of SMA and reduced the expression of mTOR. This suggests that mTOR is involved in the crosstalk between the injured stimuli and metabolic demand to regulate the phenotype of the VSMCs.

mTOR, the key downstream target of PDGF, is a member of conserved serine/threonine kinase. Since mTOR plays an important role in metabolic regulation, many mTOR-inhibiting strategies have used to inhibit the VSMCs phenotypic switch [15]. However, recent studies have demonstrated that a phenotypic switch from the contractile type to synthetic type can be attributed to activated autophagy, although the expression of mTOR is upregulated [16]. Interestingly, the treatment of rapamycin or rapamycin-based drug-eluting stents still promotes a contractile phenotype [15,17,18]. Consistent with this study, our results demonstrate that the inhibition of the PDGF-induced VSMCs’ phenotypic switch by BP is associated with the inhibition of mTOR. Besides, we observed that LC3-II, which is an indicator of activated autophagy, was also inhibited. These data suggest that, in the VSMCs, the regulation of phenotypic switch is mTOR dependent although the relation between mTOR and autophagy remains controversial. Because VSMCs are highly plastic and can quickly adapt their phenotype in response to environmental stimuli, the intracellular ion concentration of VSMCs is likely to be involved in the phenotypic switch and autophagy. According to previous studies, the autophagy can be inhibited by cytosolic Ca^2+^ [19]. In addition, the treatment of rapamycin increases the cytosolic Ca^2+^ [20], indicating that the regulation of cytosolic Ca^2+^ by mTOR may determine the phenotype and autophagy of VSMCs. Hence, another possible explanation is that the treatment of BP may result in an increase of cytosolic Ca^2+^ in an AMPK–mTOR-dependent manner, thereby inhibiting the autophagy and phenotypic switch of the VSMCs. Since the present study has only examined the effects of BP in the phenotypic modulation, future studies are needed to examine the cytosolic Ca^2+^, autophagy and their interactions.

Taken together, our findings demonstrate that BP serves as an AMPK activator, which can inhibit mTOR and promote VSMCs toward the differentiated, quiescent contractile type from a metabolic aspect. Hence, BP might be a novel promising compound to be used against vascular disorders such stenosis and restenosis.

## 4. Materials and Methods

### 4.1. Cell Culture

The rat aortic smooth muscle cell A7r5 was purchased from the Bioresource Collection and Research Center (BCRC, Hsin Chu, Taiwan). Cells were cultured in Dulbecco’s Modified Eagle Medium (Hyclone, Logan, UT, USA), consisting of 10% fetal bovine serum (Gibco, Thermo Fisher Scientific, Waltham, MA, USA) at 37 °C in a humidified atmosphere containing 5% CO_2_. For the subsequent in vitro study, we also used Dulbecco’s modified eagle medium (DMEM) consisting of 10 ng/mL PDGF-bb as the control.

### 4.2. Antibodies and Reagents

The following antibodies used for Western blotting are listed: anti-GAPDH (MAB374, Merck Millipore, Billerica, MA, USA), anti-alpha SMA (ab5694, Abcam, Cambridge, UK), anti-vimentin (5741, Cell Signaling, Beverly, MA, USA), anti-phosphorylated AMPK (2535, Cell Signaling), anti-AMPK (2532, Cell Signaling), anti-phosphorylated mTOR (5536, Cell Signaling), anti-mTOR (ab32048, Abcam) and anti-collagen I (sc-166865, Santa Cruz, CA, USA). The chemicals used were PDGF-bb (520-BB, R&D System, Minneapolis, MN, USA), PDGF-bb(520-BB, R&D System) and n-butylidenephthalide (ALFA, Derbyshire, UK). For the in vitro experiments, 10% (*w*/*v*) n-BP/DMSO was prepared as the stock.

### 4.3. Migration Assay

Both a traditional wound healing assay and Oris migrating assay were performed to address the migrating ability of smooth muscle cells [12]. The A7r5 cells were seeded in 6- and 96-well plates and cultured until 80% confluent. Then, the culture medium was replaced by DMEM containing 10 µg/mL mitomycin C (Sigma-Aldrich, St. Louis, MO, USA) for 30 min to inhibit mitosis. For the wound healing assay, a straight wound was created via scraping on the 6-well plate by a sterile tip; for the Oris migrating assay, the stoppers were then removed. Both the 6-well and 96-well plates were washed by phosphate-buffered saline to remove floating cells and were incubated with DMEM, which consisted of 10 ng/mL PDGF (R&D System) and BP at various concentrations. The migrating ability was then measured under microscope and analyzed at 24 h. The values were expressed as the migration percentage as compared to the starting point.

### 4.4. Cell Cycle Analysis

The distribution of the VSMCs in different cycle phases was performed by flow cytometry after PI staining. In brief, VSMCs were grown in 6-cm cell culture dishes and treated with PDGF in the presence or absence of BP for 24 h. The cells were then collected, washed by phosphate buffered saline (PBS) and fixed with 10% neutralized formalin. Next, the PI staining was performed and then analyzed by a flow cytometer (FC500, Backman Coulter, Brea, CA, USA).

### 4.5. Immunofluorescence

The A7r5 cells were seeded on 4-well chamber slides at 500 cells/well density. After the confluence reached 50%, the medium was replaced by DMEM consisting of 10 ng/mL PDGF and BP at various concentrations for another 24 h. The cover of the chamber slide was then removed. Cells were fixed with 10% neutralized formalin for 10 min and washed with PBS. The staining step was performed by 5% bovine serum albumin blocking followed by incubating with the primary antibody at 4 °C overnight. A fluorescence-conjugated secondary antibody was then applied for 1 h, followed by PBS washing and Hoechst33342 staining for 15 min. Eventually, the slides were mounted and then observed under a fluorescence microscope (Olympus, IX70, Tokyo, Japan).

### 4.6. Western Blot Analysis

The total protein was extracted by 1X RIPA buffer after the A7r5 cells were treated by PDGF and variable concentrations of BP. The quantification of total protein was performed by a Pierce 660 nm Protein Assay (Thermo Scientific, Waltham, MA, USA). A total of 10 µg of denatured-reducing protein was applied to electrophoresis on a polyacrylamide gel and then transferred to polyvinyldenefluoride membranes. A total of 5% BSA was used to block nonspecific binding followed by being incubated with primary antibodies at 4 °C overnight. Membranes were washed and incubated with an HRP-conjugated secondary antibody for 1  h at room temperature. The chemiluminescence was excited by ECL reagent, and the intensity of the signal was detected by an iBright CL1000 Imaging System (Thermo Scientific).

### 4.7. Transient Transfection of siRNA

Transient transfection of siRNA was performed using Lipofectamine 2000 (Invitrogen, Waltham, MA, USA) according to the manufacturer’s instructions. For gene knockdown, A7r5 were grown to 40% confluence and treated with PDGF and BP for 24 h. Cells were then transfected with the 10 μM control or AMPK siRNA. After a 48-h incubating period, the experiments were performed.

### 4.8. Animal Model

The surgical creation of a peripheral AVF for this study was performed in Sprague Dawley (SD) rats weighing 225 to 275 g using an end-to-side anastomosis of the femoral artery to the femoral vein according to a previous study [21]. Sixteen adult male SD rats receiving AVF were assigned into four groups randomly and were treated with oral application of BP at 0 mg/kg-QD (canola oil control), 20 mg/kg-QD, 50 mg/kg-QD or 50 mg/kg-BID dosage. The 0%, 2% and 5% of BP/canola oil (*w*/*v*) were prepared so that the feeding volume can be determined by the weight of the rats. All these rats were sacrificed on Day 30. The venous limbs of the AVF were fixed and embedded in paraffin. Cross sections (3 μm) were prepared to apply regular and pathological staining. All animal experiments were in accordance with the Institutional Animal Care and Use Committee and under the permission number 004.

### 4.9. Immunohistochemical Staining

The paraffin sections underwent a programmed deparaffinization and rehydration procedure through xylene and a graded series of ethanol solutions. Heat-induced antigen retrieval was performed followed by incubating in 3% hydrogen peroxide to quench endogenous peroxidase activity. The sections were incubated by 5% bovine serum albumin for 1 h at RT to inhibit nonspecific binding. The primary antibody was incubated at 4 °C overnight. The HRP-conjugated secondary antibody and DAB staining method were then applied according to the manufacturer’s protocols. The sections were counterstained with hematoxylin and mounted. The digital photos were taken using an IX70 (Olympus) microscope equipped with a CCD.

### 4.10. Statistical Analysis

All statistical comparisons were completed by the two-tailed Student’s *t*-test in Microsoft Excel and GraphPad Prism 7. Data are summarized as the mean ± standard deviation. A *p* value < 0.05 was considered significant.

## Abbreviations

BP	N-butylidenephthalide
VSMCs	Vascular smooth muscle cells
PDGF	Platelet-derived growth factor
AMPK	5′ AMP-activated protein kinase
mTOR	Mammalian target of rapamycin
AVF	Arteriovenous fistula
